# Adolescents’ and Parents’ Perspectives of a Revised Protein-Sparing Modified Fast (rPSMF) for Severe Obesity

**DOI:** 10.3390/ijerph16183385

**Published:** 2019-09-12

**Authors:** Keeley Pratt, Jennifer Cotto, Jinyu Xu, Rosanna Watowicz, Marnie Walston, Ihuoma Eneli

**Affiliations:** 1 Department of Human Sciences, The Ohio State University, 130 Campbell Hall, 1787 Neil Avenue, Columbus, OH 43210, USA; 2 Department of Surgery, The Ohio State Wexner Medical Center, Columbus, OH 43210, USA; 3 Center for Healthy Weight and Nutrition, Nationwide Children’s Hospital, Columbus, OH 43210, USA; 4 Department of Nutrition, Case Western University, Cleveland, OH 43210, USA; 5 Department of Pediatrics, Akron Children’s Hospital, Akron, OH 43210, USA; 6 Department of Pediatrics, The Ohio State University, Columbus, OH 43210, USA

**Keywords:** adolescents, parents, protein-sparing modified fast, weight management

## Abstract

The purpose of this pilot study was to assess the acceptability to adolescents (11–18 years old) and their parents using a revised protein-sparing modified fast (rPSMF) for 12 months as an intervention for severe obesity in a tertiary pediatric weight management clinic (PWMC). To assess acceptability (satisfaction, confidence) with the rPSMF protocol, surveys were completed by adolescents and parents at baseline, 1, 3, 6, and 12 months, with adolescent height and weight measured. Analyses were conducted to assess differences in satisfaction and confidence based on adolescent response (weight loss) and adherence to the rPSMF. Adolescents’ adherence with the rPSMF was close to 50% in the first 3 months, but dropped to 14.7% at 6 months. Adolescents were most confident with choosing low carbohydrate foods at baseline. Over 12 months, adolescents and parents reported weight loss as “the most liked” part of the rPSMF. Adolescents who were adherent were more satisfied with their weight loss than their non-adherent peers. Parents and adolescents reported lack of food variety and difficulty following the protocol as challenges with the rPSMF. Adolescents and their parents were able to follow the rPSMF protocol, with weight loss, but with decreased adherence over time.

## 1. Introduction

It is well established that obesity in youth is an epidemic, with 1 in 5 youth ages 2–19 in the United States (US) experiencing obesity [1]. Adolescents are particularly affected, as 41.5% of 16–19 years-olds have an overweight or obese weight status, and 4.5% are classified with severe obesity (Body Mass Index (BMI) ≥ 140% of the 95^th^ percentile) [1]. Despite public health initiatives aimed at preventing obesity and rigorous treatment guidelines, the prevalence of severe obesity remains steady or slightly increased among youth of all ages [1,2,3]. This has led to calls for treatment options that extend beyond conventional pediatric weight management lifestyle interventions [4,5,6,7].

Standard pediatric weight management for youth with obesity involves lifestyle interventions delivered by a multidisciplinary team during weekly or monthly visits at outpatient clinics or community programs. However, youth with severe obesity respond poorly to this type of treatment when compared to their peers with overweight and obesity [8]. Bariatric surgery, a more intensive treatment option, has risen in popularity and acceptability for adolescents with severe obesity; yet, despite the large number of eligible candidates, access to bariatric surgery for adolescents is limited [9,10].

An alternative non-surgical option that has produced promising weight loss results among adults and in select studies of youth with obesity [11,12,13,14,15,16,17,18,19] is the protein-sparing modified fast (PSMF), a reduced calorie, low-carbohydrate, high protein dietary approach. A hallmark of the PSMF is the limited carbohydrates available for metabolism that drives the liver to oxidize fatty acids in order to produce energy [20]. During the process of fatty acid oxidation, ketones are produced [21]. Fatty acid oxidation is protective for typical appetite-regulating hormone shifts that occur with conventional reduced-calorie dietary approaches, resulting in appetite suppression [22,23]. The use of fatty acids as an energy source also leads to greater fat loss through the catabolism of adipose tissue in the body. Protein in the PSMF dietary approach is “spared” or maintained at normal levels to protect against the loss of lean muscle mass [11,12,13,14,15,16,17].

Despite studies noting the safety, efficacy, and resulting weight loss among adolescents using the PSMF dietary approach, the PSMF is not a standard treatment option for adolescents with obesity [11,12,13,14,15,16,17]. In part, there is not extensive extant literature on the use of PSMF in adolescents with obesity, and published studies have only used the PSMF diet over a short duration (e.g., ≤3 months) [11,12,13,14,15,16,17]. Because fostering ketosis (production of ketones) for weight loss in adolescents with obesity is an aggressive intervention, it is important to understand the experiences of both adolescents and caregivers/parents (referred to parents herein) with using the PSMF dietary approach as a treatment for adolescent obesity. To date, no study has investigated the perspectives or experience of these adolescents and their parents while participating in a PSMF. Obtaining perspectives from adolescents and parents will provide insight into the strengths of the PSMF dietary approach, as well as challenges to address to improve the future acceptability and feasibility of the PSMF dietary approach as a viable non-surgical treatment option for adolescents with obesity. The purpose of this pilot study was to assess the acceptability of using the revised protein-sparing modified fast (rPSMF) over 12 months among adolescents (11–18 years-old) and their parents as an intervention for severe obesity in a tertiary pediatric weight management clinic (PWMC). Acceptability assessments included adolescents’ and parents’ perceptions of the benefits, level of satisfaction and challenges with their use of the rPSMF for severe obesity.

## 2. Materials and Methods

### 2.1. Participants and Recruitment

Adolescents recruited for the study were seen routinely for their standard care visits in an outpatient Stage 3 [2] PWMC, which provides care to youth and adolescents with a BMI above or equal to the 95th percentile. If the adolescent met the major inclusion criteria (see Table 1 [24]) for an intensive, restricted dietary option, the medical provider discussed the use of a liberalized rPSMF of approximately 1200–1800 kcal/day, in addition to other appropriate treatment options available in the center (e.g., behavioral lifestyle intervention, low glycemic load diet, bariatric surgery). After the adolescent, parent, and the medical provider agreed to use the rPSMF diet, the research coordinator had the adolescent and parent sign assent and consent forms, and enrolled them into the study. An adolescent may have decided to go on the rPSMF but not participate in the study, but an adolescent could not be enrolled in the study if the rPSMF was not their agreed upon treatment plan.

Adolescent Class II (BMI > 120% of the 95th percentile, or BMI ≥ 35) or Class III obesity (BMI ≥ 140% of the 95th percentile, or BMI ≥ 40) was the primary criterion for participation in the rPSMF [1]. The inclusion criteria were stricter for younger (11–13 years old) adolescents, who had to have at least one severe comorbidity (i.e., obstructive sleep apnea, type II diabetes, fatty liver, slipped capital femoral epiphysis, Blount disease, pseudotumor cerebi), while the older adolescent group only had to have one non-severe comorbidity. Exclusion criteria for both age groups included the diagnosis of cardiac arrhythmia, lack of insurance coverage, impairment of renal function, positive pregnancy test, and abnormal lab values, including elevated creatinine (>0.9 mg/dL or Glomerular Filtration Rate (GFR) < 90 mL/min/1.73 m^2^), baseline uric acid or thyroid stimulating hormone (TSH). Because this was a pilot study to determine feasibility of recruitment and acceptability of the rPSMF, a formal sample size calculation was not conducted [25].

### 2.2. Protocol

The protocol for the rPSMF used at the PWMC has been described in prior work [24]. Adolescents were seen biweekly for the first eight weeks—a period termed the “ramp-up phase”—then monthly for the remainder of the 12 months by a multidisciplinary team. More frequent visits were scheduled as needed. The team included a medical provider, dietitian, physical therapist, and psychologist and social worker as needed. The rPSMF was provided through three phases. Phase 1 (baseline through 6 months) was the most restrictive, allowing for 40 g of carbohydrates per day. During Phase 2 (7 through 12 months), daily carbohydrate intake was increased to 60 g per day through the introduction of fruits and low-fat dairy products. Finally, in Phase 3 (at 12 months), carbohydrate intake was gradually increased to a set point of approximately 100–200 g per day. Daily protein intake was 1.2–1.5 g of protein per kilogram of ideal body weight, with an emphasis on low fat and lean protein options. Meal replacement drinks or supplements were not specifically recommended or prohibited. Calorie intake ranged from 1200–1800 calories/day based on adolescents’ age, estimated ideal body weight, and level of physical activity. A daily intake of 2–3 L of fluid was recommended, and daily multivitamin, calcium supplement, and vitamin D supplements were prescribed for the duration of the intervention. Each adolescent and parent dyad worked with a dietitian to create meal plans for several days using a food preference sheet and shopping list.

### 2.3. Evaluation of the rPSMF

This prospective observational study of the rPSMF treatment protocol was approved by the Hospital’s Institutional Review Board (IRB15-00667). Adolescents and parents completed assessments about their satisfactions and progress with the rPSMF, height and weight, and physical activity at baseline and at 1, 3, 6, and 12-month follow-up. Adolescents and parents each received compensation for completing assessments at each time point. The assessments were administered using REDCap (Research Electronic Data Capture, Vanderbilt University, Nashville, TN, USA), a Health Insurance Portability and Accountability Act (HIPAA)-adherent online survey software. Standard clinical demographics were collected at baseline from adolescents and parents, including race/ethnicity, gender, insurance status, and adolescent date of birth. Parents reported their height and weight at baseline, which was used to calculate their BMI.

The adolescent’s height and weight was measured at each follow-up visit and was used to calculate BMI, BMI percentile, and BMI z-score. Raw BMI was calculated using height in meters squared divided by weight in kilograms; BMI percentile and BMI z-score were also calculated based on adolescent BMI, age, and gender [26,27]. BMI z-scores show the standard deviation of a child’s BMI from the average BMI of a same age and gender group [16]. Change in weight, BMI, percentage of the 95th percentile for BMI (%BMIp95), and BMI z-score from baseline were calculated at 1, 3, 6 and 12 months [27]. In the analysis, weight loss was used as a dichotomous variable, adolescents either lost weight or gained weight/stayed the same weight determined by change in their weight from baseline to each follow-up visits (1, 3, 6, and 12-months). Weight, rather than BMI z-score and %BMIp95, was used as it is a more tangible and meaningful measure to pair with acceptability of the rPSMF for adolescents and parents.

Adherence with the rPSMF was determined by the registered dietitian (RD) at each clinic visit using 24-h dietary recalls and food frequency questionnaires. If adolescents were able to consume 40 ± 10 g of carbohydrates per day with weight loss over the past month, adolescents were considered “adherent”. If the adolescent noted attempts to consume low carbohydrate meals and/or snacks throughout the past month, but did not always reach the goal of 40 ± 10 g of carbohydrates per day with weight loss, they were considered “moderately adherent”. If the adolescent reported none or very few attempts to consume low carbohydrate meals and/or snacks with weight gain or maintenance within the past month, they were considered “not adherent”. For the analysis, adherence was categorized as a dichotomous variable where adolescents were noted as “not adherent” or “adherent” by combining the moderately adherent and adherent categories.

The survey questions were based on extant literature on PSMF approaches and the team’s prior experience using the rPSMF in the PWMC. The questions assessed adolescents’ and parents’ (1) overall confidence following the rPSMF (choosing low carbohydrate foods, locating the carbohydrate content on food labels, following meal plans provided by dietitians, checking adolescents’ urine for ketones), (2) satisfaction with weight loss, weight changes, and the rPSMF; and asked open-ended questions about the acceptability of the rPSMF. Table 2 outlines the questions, responses options, and assessment intervals.

### 2.4. Analysis

The Likert scale questions for confidence in Table 2 were analyzed using descriptive statistics for adolescents and parents at each time point in the study. Likert scale options were converted to numeric values to determine mean scores for adolescent and parent groups at each time point: Strongly Agree (4), Agree (3), Disagree (2), and Strongly Disagree (1). Differences in confidence and satisfaction questions based on adolescent weight loss status (lost weight, gained weight/stayed the same) and adherence with rPSMF (adherent, non-adherent) were assessed using independent *t*-tests. Differences between adolescents’ and parents’ responses at each time point on the satisfaction questions (ranging from 0–100) were assessed using paired *t*-tests.

The open-ended questions were analyzed at the case level, where text was coded for each unique case (adolescent or parent) response within each question at baseline, 1, 3, 6, and 12 months. Text was coded independently by two coders (KP and JX) for similar responses or themes within each question. When discrepancies arose, a third coder (JC) was triangulated to resolve the discrepancy. For each question, the frequency and percent of unique responses for each theme with at least 15% of total cases were presented. Of note, the frequencies and percentages listed in each question do not add up to the total number of respondents or 100%, since the frequency and percentage of case respondents was calculated for each theme within each question.

## 3. Results

Over a 12-month period, 65 adolescents and their parents were identified as potential candidates for the study based on the inclusion criteria and were offered the rPSMF as a treatment option. Twenty-nine (44.6%) of those adolescents progressed to the ramp-up phase. Twenty-one (32.3%) successfully completed the ramp-up phase, started the rPSMF, and enrolled in the study. The demographics of those enrolled in the study and those who did not enroll in the study were similar [28]. Two adolescents who enrolled in the study were twins with one shared parent between them. A number of eligible adolescents and parents declined to participate in the rPSMF because they felt the meal plan was too repetitive and/or strict, were unable to keep the frequent follow up appointments, lived a far distance from the PWMC, had financial constraints, and/or had an interest in bariatric surgery or other weight management treatments. Not all adolescents or parents completed the survey at each follow-up point, and there were occasions when an adolescent and/or parent may have missed one follow-up point (for example, month 3), but provided data for a later follow-up point (for example, month 6). Due to the small sample size, if adolescents and/or parents did not complete the survey at one follow-up point but remained in the study and provided survey data at the next follow-up point, they were included in the analysis. For the analysis, 21 adolescents (100%) provided completed data at baseline, 20 (95.2%) at 1 month, 19 (81.0%) at 3 months, 18 (71.4%) at 6 months, and 17 (61.9%) at 12 months. For the parents, 20 (100%) provided completed data at baseline, 18 (90%) at 1 month, 17 (85%) at 3 months, 17 (85%) at 6 months, and 17 (85%) at 12 months.

### 3.1. Adherence and Anthropometric Outcomes

The baseline characteristics of adolescents and parents are described in Table 3. There were no significant differences based on demographics (race/ethnicity, gender) between those who dropped out and those who remained in the study. Adherence with the rPSMF declined over the 12 months. At 1 month, 50.0% (10/20) of the adolescents were adherent, 47.4% (9/19) at 3 months, 16.7% (3/18) at 6 months, and 11.8% (2/17) at 12 months. From baseline, at 1 month, 90.0% (18/20) of the adolescents lost weight, 78.9% (15/19) of the adolescents lost weight at 3 months, 72.2% (13/18) of the adolescents lost weight at 6 months, and 35.3% (6/17) of the adolescents lost weight at 12 months. Regardless of adherence to the rPSMF, the mean weight change was −3.7 ± 3.5 kg, (range −13.5 to 0.9 kg) at 1 month, −5.5 ± 5.1 kg, (range −19.3 to 1.8 kg) at 3 months, −4.7 ±6.6 kg, (range −18.3 to 8.6 kg) at 6 months and −1.3 ± 10.6 kg, (range −17.7 to 14.8 kg) at 12 months. Adolescents who were adherent to the rPSMF lost more weight than non-adherent adolescents, with statistical significance at 3 months (−8.0 ± 5.5 kg vs. −3.2 ± 3.5 kg, *p* = 0.04) [28].

### 3.2. Confidence

**Figure 1:**
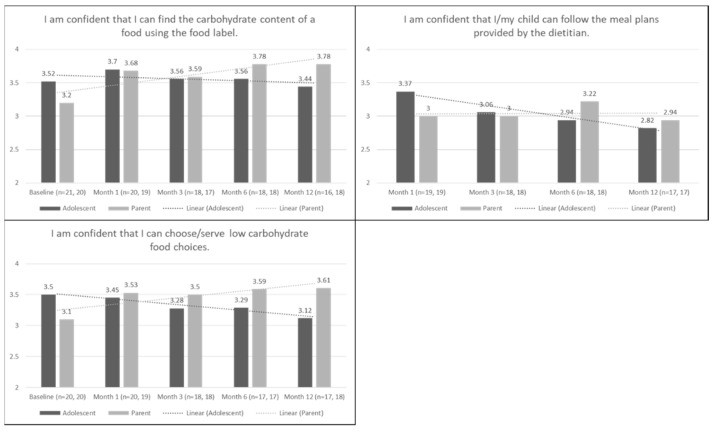
Adolescent and parent mean trends in confidence over 12 months.

Figure 1 shows the mean scores of both adolescent and parent reports on the four confidence questions at baseline (when applicable) and follow-up. For the question “I am confident that I can find the carbohydrate content of a food using the food label”, from baseline to 12 months adolescent mean scores tended to slightly decrease, whereas parent mean scores slightly increased. At baseline, adolescents had greater confidence in finding the carbohydrate content of a food using the food label compared to parents, however at 12 months parents became more confident than adolescents. Similarly, from baseline to 12 months, on the statement “I am confident that I can choose/serve low carbohydrate food choices”, adolescents and parents had mean scores between 3–4, where again adolescent mean scores decreased and parent mean scores increased. For the statement “I am confident that I/my child can follow the meal plans provided by the dietitian”, adolescent and parent scores were between 2–3 (disagree to agree), in which adolescent scores decreased and parent scores were stable from 1 month to 12 months. For the parent-only statement “I am confident that I can buy a variety of lean protein food options for meals and snacks,” scores increased consistently over 12 months.

### 3.3. Confidence Based on Adherence and Response (Weight Loss) to the rPSMF

Adolescent and parent answers to the confidence questions based on adolescent adherence (adherent versus non-adherent) and response to the rPSMF (lost weight or gained weight/maintained weight) varied at different time points during the intervention.

(a) One month: There were no significant differences in confidence ratings based on adolescent adherence to the rPSMF at one month, though there was a significant difference in confidence rating based on response to the rPSMF at one month. Adolescents who lost weight on the rPSMF from baseline to 1 month (*n* = 17, Mean (M) = 3.47, Standard Deviation (SD) = 0.51) reported higher confidence in following the meal plans provided by the dietitian as compared to adolescents who did not lose weight (*n* = 2, M = 2.50, SD = 0.71; *t*(17) = 2.46, *p* = 0.025).
(b) Three months: There were no significant differences in adolescent reports or parent reports on the confidence questions based on whether the adolescent was adherent with the rPSMF approach at 3 months, and there was not a large enough sample (weight loss *n* = 15, weight gain/stayed the same *n* = 1) at three months to conduct analyses based on response to the diet.
(c) Six months: Adolescents who were adherent (*n* = 3, M = 4.00, SD = 0.00) had greater confidence in reading food labels than adolescents who were not adherent (*n* = 12, M = 3.58, SD = 0.67; *t*(11) = −2.16, *p* = 0.054). There were no significant differences for adolescent and parent responses on the confidence questions based on whether adolescents lost weight or gained weight/stayed the same.
(d) Twelve months: Parents of adolescents who were adherent (*n* = 2, M = 4.00, SD = 0.00) had greater confidence in reading food labels than parents of adolescents who were not adherent (*n* = 9, M = 3.56, SD = 0.53, *t*(8) = −2.53, *p* = 0.035). Parents of adolescents who were adherent (*n* = 2, M = 4.00, SD = 0.00) also reported greater confidence in serving low carbohydrate food choices than parents of adolescents who were not adherent (*n* = 9, M = 3.56, SD = 0.53; *t*(8) = −2.53, *p* = 0.035). Parents of adolescents who were adherent (*n* = 2, M = 4.00, SD = 0.00) reported greater confidence that their child could follow the meal plans provided by the dietitian compared to parents of adolescents who were not adherent (*n* = 8, M = 2.88, SD = 0.35; *t*(8) = −4.30, *p* = 0.003). There were no significant differences for adolescent and parents’ reports of confidence based on adolescents who lost weight or gained weight/stayed the same.

### 3.4. Satisfaction

The descriptive statistics for the satisfaction questions for both adolescents and parents are in Table 4. For both adolescents and parents, their mean scores for satisfaction with weight loss and weight changes decreased over time; the mean scores for difficulty using the rPSMF were stable over 12 months.

(a) Adolescent and Parent Changes over Time: There was a significant difference in response from 1 month to 12 months for adolescents (*n* = 16) and parents (*n* = 17) to the question “How much do you feel that the low-carbohydrate, high-protein diet has helped you/your child lose weight?” Adolescents at 1 month (M = 84.75, SD = 15.18) felt that the rPSMF approach helped them to lose weight more than they reported at 12 months (M = 65.44, SD = 21.26; *t*(15) = 2.90, *p* = 0.011). Parents also had higher reports at 1 month (M = 86.06, SD = 22.30) than 12 months (M = 63.24, SD = 29.56; *t*(16) = 3.64, *p* = 0.002).

There was a trend to significance for adolescents (*n* = 13) and a significant difference for parents (*n* = 17) in response to the question “How satisfied are you with your weight change so far?” between 1 month and 12 months. Adolescents had higher reports of satisfaction with weight changes at 1 month (M = 68.46, SD = 24.83) than 12 months (M = 50.62, SD = 26.71; *t*(12) = 2.04, *p* = 0.064). Similarly, parents had significantly higher reports at 1 month (M = 81.06, SD = 21.44) compared to 12 months (M = 58.65, SD = 29.55; *t*(16) = 2.31, *p* = 0.035).

(b) Adolescent-Parent Comparisons over Time: See Table 4 for comparisons between adolescents’ and parents’ mean scores on the satisfaction questions at each time point. Overall, on the question, “How much do you feel that the low-carbohydrate, high-protein diet has helped you/your child lose weight?”, parents had higher satisfaction with weight loss at each follow-up time point than adolescents. However, the difference between adolescents and parents scores became smaller over time, meaning they became more congruent in their perceptions; 1 month mean difference = 9.00 (SD = 21.11) vs. 12 months mean difference = −1.29 (SD = 25.73). There was a significant difference at 3 months between adolescents (M = 73.00, SD = 21.98) and parents (M = 82.53, SD = 25.53), where parents significantly endorsed that the rPSMF approach helped their child lose weight (*t*(16) = −2.61, *p* = 0.016) compared to adolescents.

Similarly, for the question, “How satisfied are you with your weight change so far?” parents continued to report higher satisfaction than adolescents reported at each follow-up time point. The mean difference between adolescent and parent satisfaction with weight changes increased over time; 1 month mean difference = −8.43 (SD = 35.31) vs. 12 months mean difference = −15.19 (SD = 24.80). There was a significant difference at 6 months between adolescents and parents (M = 55.93, SD = 25.14 vs. M = 69.14, SD = 22.39; *t*(13) = −2.24, *p* = 0.043), where parents reported significantly higher satisfaction than adolescents with weight changes. The same results were found for adolescents and parents (M = 49.19, SD = 25.83 vs. M = 64.38, SD = 29.24; *t*(15) = −1.98, *p* = 0.027) at 12 months.

On the question, “How difficult has the rPSMF approach been for you to follow?”, there was no consistency in how adolescents or parents responded. For example, at 6 months adolescents (M = 3.21, SD = 31.30) reported more difficulty than parents (M = 3.47, SD = 34.22), but at 12 months, parents (M = −4.94, SD = 34.46) reported more difficulty than adolescents (M = −6.88; SD = 19.99).

### 3.5. Satisfaction Based on Adherence and Response to the rPSMF

(a) Three months: Adolescents who were adherent (*n* = 8, M = 86.50, SD = 10.89) endorsed that the rPSMF helped them lose weight more than adolescents who were not adherent (*n* = 8, M = 59.63, SD = 23.94; *t*(14) = −2.89, *p* = 0.012). Adolescents who were adherent (*n* = 8, M = 68.50, SD = 15.13) were also more satisfied with their weight changes than adolescents who were not adherent (*n* = 7, M = 46.86, SD = 18.18; *t*(13) = −2.52, *p* = 0.026). Parents of adolescents who were adherent (*n* = 8, M = 94.50, SD = 5.63) also endorsed that the rPSMF helped their child lose weight more than parents of adolescents who were not adherent (*n* = 8, M = 68.50, SD = 32.10; *t*(7.43) = −2.26, *p* = 0.057).
(b) Six months: Adolescents who were adherent (*n* = 3, M = 94.67, SD = 3.51) endorsed that the rPSMF helped them lose weight more than adolescents who were not adherent (*n* = 12, M = 64.25, SD = 29.35; *t*(12.08) = −3.49, *p* = 0.004). Parents of adolescents who lost weight (*n* = 13, M = 72.38, SD = 21.68) reported more satisfaction with weight changes compared to parents of adolescents who gained weight/stayed the same weight (*n* = 2, M = 31.50, SD = 9.19; *t*(13) = 2.56, *p* = 0.024).
(c) Twelve months: Adolescents who were not adherent (*n* = 9, M = 70.44, SD = 19.37) believed that the rPSMF was more difficult to follow than adolescents who were adherent (*n* = 2, M = 31.00, SD = 25.46; *t*(9) = 2.51, *p* = 0.034). Similarly, parents of adolescents who were not adherent (*n* = 8, M = 73.25, SD = 17.50) believed that the rPSMF was more difficult to follow than parents of adolescents who were adherent (*n* = 2, M = 25.00, SD = 35.36; *t*(8) = 2.96, *p* = 0.018). Adolescents who lost weight (*n* = 7, M = 58.86, SD = 27.38) reported more satisfaction with weight changes compared to adolescents who gained weight/stayed the same weight (*n* = 4, M = 21.50, SD = 16.09; *t*(9) = 2.46, *p* = 0.036). Parents of adolescents who lost weight (*n* = 7, M = 82.14, SD = 21.05) endorsed that the rPSMF helped their child lose weight more than parents of adolescents who gained weight/stayed the same weight (*n* = 4, M = 32.50, SD = 16.62; *t*(9) = 4.02, *p* = 0.003).

### 3.6. Open-Ended Questions

Table 5 outlines the themes, frequency and representative examples of responses in quotations. Most adolescents and parents reported the outcome that they liked the most was “weight loss”. Other specific things that adolescents endorsed as liking were food specific to the rPSMF (“food taste”, “food variety”, and “trying new foods”). The things that parents noted liking were that the rPSMF was “easy to follow”, the “food variety” and “family involvement” with the rPSMF. Most parents reported that they “buy low carb foods and limit low carb options in the home” for the whole family. They also identified that they “make healthier nutrition choices” (*n* = 6, 30%) by reading food labels, limiting portion size and limiting energy-dense foods. A number of adolescents reported that the rPSMF approach was “hard to follow” and some parents noted the rPSMF approach was “too restrictive”. The most common difficulty noted by both adolescents and parents was the lack of variety in food choices and restriction of carbohydrates.

## 4. Discussion

The purpose of this pilot study was to evaluate the acceptability of the rPSMF intervention among adolescents and their parents in a Stage 3 PWMC over 12 months. Obtaining both adolescents’ and parents’ perspectives of acceptability provides additional understanding into the process of behavior change, barriers to adoption of the rPSMF, and factors that affect adolescent and parent adherence to the rPSMF in the short and long term. Overall, despite limited adherence to the rPSMF, most adolescents in the study still experienced declines in BMI z-score. Given the lack of response to traditional treatment for adolescents with severe obesity [2,3], this is an important outcome which may imply that a rPSMF, even in the absence of full adherence, is a viable treatment alternative for adolescent weight loss [28]. Although poor adherence is a common challenge in pediatric weight management [29,30], the highly structured nature of the rPSMF may have challenged adolescents and parents to pay more attention to their food selection, leading to reduced calorie intake and weight loss, even in the absence of full adherence.

The majority of adolescents and parents in the study reported moderate confidence with several aspects of the rPSMF including reading food labels and choosing low carbohydrate foods, but less confidence following the meal plans provided by the dietitian. Parents tended to be more confident than their adolescents, with the exception of their baseline assessment, and their confidence increased over 12 months. Whereas, adolescents were more confident than their parents were at baseline, but their confidence decreased on all questions throughout the 12 months. Discrepancies between parent and adolescent reports of behavior change and weight-related outcomes have been reported in prior pediatric weight management research; however, in prior work parent reports tend to be less optimistic than adolescent reports [31,32]. Specific to this study, parents’ increased confidence may have been affected by observing adolescents’ behavior change. Since the rPSMF has very clear guidelines to follow, if adolescents were selecting low-carbohydrate, high-protein foods at home, this may have been observed by their parents. Skelton and colleagues also noted that parents desired highly structured programs with clear observable behavioral goals to work towards [30]. Adolescents may have had less support in following the rPSMF amongst their peers and in social situations away from their parents, hence, lower adolescent confidence with the rPSMF. Future research should explore if adolescents have more trouble selecting foods consistent with the rPSMF when they are away from home or eating separately from their parents.

Adolescent weight loss and adherence also appears to play a role in both adolescents’ and parents’ reports of confidence in following the rPSMF. At 1 month, adolescents who had lost weight from baseline reported being more confident in following the meal plans provided by the dietitian compared with adolescents who had not lost weight. Confidence with following the meal plans may have led adolescents to follow the meal plans, and subsequent weight loss. Additionally, early weight loss in treatment is a strong predictor for subsequent and sustained weight loss. For example, Unick and colleagues conducted a randomized controlled trial of over 5000 adults, and found that early weight loss within the first two months in an intensive lifestyle intervention was predictive of subsequent weight loss and maintenance over eight years [33]. Perhaps early weight loss while using the rPSMF approach is critical for adolescents to gain confidence with the rPSMF.

Interestingly, at 12 months, parents of adolescents who were adherent to the rPSMF reported more confidence on all questions compared to parents of adolescents who were not adherent. Reading food labels for carbohydrate content, choosing low-carbohydrate options, and following the rPSMF meal plans were skills that the dietitians worked on with adolescents and parents at each visit. Thus, adolescents showing adherence with the rPSMF possibly had improved or mastered this knowledge, which parents likely observed. Adherence may be a better indicator of parental confidence in the rPSMF than their adolescent’s observable weight loss. Similarly, Theim and colleagues observed that youth who reported higher adherence to dietary recommendations had better two-year weight-related outcomes [34]. Future research should consider looking at what determines or predicts confidence with adherence in restrictive dietary interventions and its relationship to short and long-term weight-related outcomes.

Overall, both adolescents’ and parents’ reports of satisfaction with weight changes and weight loss decreased over 12 months. This may have been affected by the pattern of adolescent weight loss observed, where the amount of weight loss declined over the study, even among adolescents who were adherent. Similar to the confidence questions, parents reported higher satisfaction with adolescent weight changes and weight loss compared to adolescents. However, parents and adolescents reported similar low levels of satisfaction with weight loss at the end of the study, with a steeper decline in parental satisfaction. This was not true for adolescent and parent reports of satisfaction with weight change, where parents reported significantly higher satisfaction than adolescents with weight changes throughout the 12-month period. Satisfaction with weight changes may be a more contextual question that also takes into account their expectations for weight change, rather than only objective measures of weight loss. Parental expectations may have been more realistic. For example, any weight loss could have been viewed as a positive change by the parents, where adolescents may have expected more rapid and substantial weight loss. It is important for future studies to consider discussing weight loss expectations with both adolescents and parents to ensure realistic goals are set.

As expected, difficulty following the rPSMF was greater among adolescents who were not adherent to the rPSMF. Adolescents who were adherent reported more satisfaction with their weight loss than those who were not adherent at 3 and 6 months. At 12 months, adolescents who were non-adherent and their parents believed that the rPSMF was more difficult to follow than adolescents who were adherent and their parents. This study only assessed satisfaction with weight changes and weight loss and difficulty following the rPSMF. Future research should include additional formal satisfaction measures to assess the rPSMF in more detail, including patient characteristics, psychosocial factors, and expectations of different aspects of treatment [30,35].

Further reinforcing the relationship between lowered confidence and satisfaction in the absence of significant weight loss, both adolescents and parents reported adolescent “weight loss” as the most frequently liked aspect of the rPSMF. They also disliked “limiting carbohydrates”, “lack of food variety”, and the overall rPSMF being “hard to follow”. Specific things that made the rPSMF difficult to follow for adolescents included “not being able to eat foods others in family are (eating)” and “eating with peers”. Parents reported both “time” and “cost as factors making the rPSMF difficult to follow. Addressing aspects that parents and adolescents disliked or were dissatisfied with is important. Most of the extant literature in pediatric weight management focuses on satisfaction only [29], as it is likely that dissatisfaction may be associated with attrition or drop out from treatment. Incorporating ways for adolescents to address their concern about being around family and peers while on the rPSMF may aid adolescents in navigating potential social situations where they feel ostracized because of their food choices.

### Limitations

The results need to be interpreted with care as to not generalize adolescents’ and parents’ responses in this study as a definitive description of acceptability and response to the rPSMF. Most participants were older adolescents and their experience may not adequately reflect those of younger adolescents who have less independence around eating or food choices. We also had one set of twin siblings with a shared parent included in our analysis. Since this was a pilot study, we included the sibling pair, however their shared genetics and home environment likely contributed to their similar responses. Adherence to the rPSMF was based on adolescents’ self-reported 24-h dietary recall and responses to food frequency questionnaires, which may be biased due to the likelihood of giving socially desirable responses. Objective measures (i.e., urinary ketones) could provide less-biased categorization in future studies. Although data were collected over a 12-month period providing a large and robust aggregate of data, the sample size was small with substantial attrition over the 12 months. At the onset of treatment in the PWMC, adolescents were presented the opportunity to enroll in the rPSMF or to choose another weight management option such as bariatric surgery, which could create selection bias. At the time when the rPSMF was introduced to adolescents and parents, there were no significant differences between those who chose to enroll in the study based on adolescents’ weight status or dietary behaviors. However, there may have been other demographic characteristics or behaviors that were not assessed that may have distinguished those who chose to enroll. Finally, the lack of a comparative population on a different specialized or restrictive diet using a randomized study design limits our ability to confidently ascertain that the findings were due to the rPSMF.

## 5. Conclusions

It is important to understand adolescents’ and parents’ satisfaction with the rPSMF to ensure that this approach meets their expectations, weight loss goals are realistic, and that families are set up for success with respect to adherence. All these factors have been shown to contribute to attrition from pediatric weight management programs [29,36]. To our knowledge, this is the first study to explore perceptions from adolescents and parents about implementing a restrictive diet like the rPSMF in an outpatient PWMC. With the increased interest in specialty and low carbohydrate diets, this evaluation of acceptability of the rPSMF diet for adolescents provides insight about how adolescents and parents make decisions about a macronutrient restrictive type diet, their expectations, and challenges with adherence. Instituting a shared decision-making process between healthcare providers, adolescents, and parents with the flexibility to modify the dietary approach and objectively monitor adherence will help to address the noted challenges with confidence and satisfaction, improve desired weight loss, and enhance adherence.

## Figures and Tables

**Table 1:** Adolescent inclusion criteria for the rPSMF [24]

	11–13 Age Group	14+ Age Group
Comorbidity	Must have severe co-morbidity: Obstructive Sleep Apnea, Diabetes (type II), Fatty Liver, Slipped Capital Femoral Epiphysis (SCFE), Blount Disease, Pseudotumor Cerebri	Must have 1 severe co-morbidity *OR* 1 co-morbidity with sustained weight gain
Pubertal Maturity	Tanner Stage III, IV, V	Tanner Stage III, IV, V
BMI	Class II or III obesity	Class II or III obesity
Rapid Weight Gain	A weight increase of more than 5% compared with baseline during the treatment; Increase in BMI z-score of 0.5 SD or more at any point during the treatment to account for age- and sex-appropriate growth	A weight increase of more than 5% compared with baseline during the treatment; Increase in BMI z-score of 0.5 SD or more at any point during the treatment to account for age- and sex-appropriate growth

BMI—body mass index; M—Mean; SD—Standard Deviation.

**Table 2:** Survey questions assessing adolescent and parent confidence and satisfaction with the rPSMF.

Question	Time Point	Who	Option Choices
Confidence Questions			
I am confident that I can find the carbohydrate content of a food using the food label.	B * F/U *	Adolescent Parent	Strongly Agree, Agree, Disagree, Strongly Disagree
I am confident that I can choose/serve low carbohydrate food choices.	B F/U	Adolescent Parent	Strongly Agree, Agree, Disagree, Strongly Disagree
I am confident that I/my child can follow the meal plans provided by the dietitian.	F/U	Adolescent Parent	Strongly Agree, Agree, Disagree, Strongly Disagree
I am confident that I can buy a variety of lean protein food options for meals and snacks.	B F/U	Parent	Strongly Agree, Agree, Disagree, Strongly Disagree
Satisfaction Questions			
How much do you feel that the low-carbohydrate, high-protein diet has helped you/your child lose weight?	F/U	Adolescent Parent	From 1 (low) to 100 (high)
How satisfied are you with your weight change so far?	F/U	Adolescent Parent	From 1 (low) to 100 (high)
How easy or difficult has this special diet been for you/your child to follow?	F/U	Adolescent Parent	From 1 (easy) to 100 (difficult)
Open-ended Questions			
So far, what have you liked about the low-carbohydrate, high-protein diet?	F/U	Adolescent Parent	Open-ended
So far, what have you not liked about the low-carbohydrate, high-protein diet?	F/U	Adolescent Parent	Open-ended
Is there anything specific that has made it difficult for you to follow the diet?	F/U	Adolescent Parent	Open-ended
Since your child has been on this diet, have you changed the way you feed the rest of your family?	F/U	Parent	Open-ended

* B—Baseline; F/U—Follow-up at all time points (1, 3, 6, 12-months).

**Table 3:** Baseline characteristics of adolescents and parents.

	Adolescent (*n* = 21) *n* (%) or Mean (SD), Range	Parent (*n* = 20) *n* (%) or Mean (SD), Range
Sex		
Male	5 (23.8%)	0
Female	16 (76.2%)	20 (100%)
Age	16.3 (1.4), 13–18	-
Race/Ethnicity		
White/Caucasian	11 (52.4%)	11 (55%)
Black/African American	9 (42.8%)	7 (35%)
Other	0 (0.0%)	2 (10%)
Hispanic	1 (4.8%)	1 (5%)
Role to Adolescent		
Mother	-	19 (95%)
Grandmother	-	1 (5%)
Baseline BMI	42.0 (6.3), 33.3–59.8	34.2 (9.8), 18.2–59.2
Baseline BMI z-score	2.5 (.27), 2.1–3.3	-
Weight Status		
Healthy weight	0	2 (10%)
Overweight	0	7 (35%)
Obese	21 (100%)	11 (55%) *

* Of the 11 parents with an obese weight status, 3 had Class I obesity, 4 had Class II obesity, and 4 had Class III obesity. M—Mean; SD—Standard Deviation.

**Table 4:** Comparison of adolescent and parent satisfaction with the rPSMF over 12 months.

	Month 1			Month 3			Month 6			Month 12		
	*n*	Mean (SD)	Range	*n*	Mean (SD)	Range	*n*	Mean (SD)	Range	*n*	Mean (SD)	Range
*How much do you feel that the low-carbohydrate, high-protein diet has helped you/your child lose weight?*												
Adolescent	19	81.21 (19.68)	26–100	18	73.00 (21.33) *	5–98	18	70.72 (26.32)	19–99	17	64.44 (20.97)	12–100
Parent	19	87.16 (21.31)	11–100	18	82.22 (24.80)	5–100	18	76.83 (24.39)	18–100	18	64.89 (29.52)	13–100
*How satisfied are you with your weight change so far?*												
Adolescent	16	64.44 (26.72)	9–100	17	58.88 (18.31)	29–94	15	56.80 (24.46) *	9–100	16	49.19 (25.83) *	0–98
Parent	18	82.11 (21.27)	17–100	18	71.61 (28.65)	6–100	17	63.71 (25.16)	25–100	18	60.72 (29.99)	15–100
*How easy or difficult has this special diet been for you/your child to follow?*												
Adolescent	16	68.13 (17.84)	19–92	17	56.18 (26.69)	1–100	17	59.41 (26.70)	2–95	17	62.00 (20.80)	13–100
Parent	16	66.44 (18.57)	31–100	18	61.72 (25.59)	6–93	16	57.94 (28.24)	0–92	17	69.35 (22.71)	0–97

* *p* < 0.05.; SD = standard deviation

**Table 5:** Adolescent and parent open-ended responses.

*n* (%)	Theme	Quote	*n* (%)	Theme	Quote
Adolescent-Liked (*n* = 20)			Parent-Liked (*n* = 20)		
11 (55.0)	Weight loss (teen)	“I like that I can see my weight loss.”	12 (60.0)	Weight loss (teen)	“It has helped him lose weight in a structured setting.”
8 (40.0)	Food taste	“The food and how good it tastes.”	10 (50.0)	Easy to follow	“Easy and manageable.”
6 (30.0)	Food variety	“It has a variety of good food options that the whole family likes.”	7 (35.0)	Food variety	“I feel that there is a variety of foods for my child to eat.”
6 (30.0)	Trying new foods	“I like the different things to try that are low carb.”	6 (30.0)	Family involvement	“It’s making the whole family aware of what foods are good and bad for you.”
6 (30.0)	Feel healthy	“It has helped me feel healthier, when I stick to it.”	5 (25.0)	Meal planning/preparation	“East to prepare….”
4 (20.0)	Easy to follow	“I don’t have to think a lot, I don’t have to count calories.”	5 (25.0)	Nutrition education	“I like to be able to watch the nutrition content.”
4 (20.0)	Protein	“Lots of meat.”	5 (25.0)	Recipes	“Easy to find recipes that she enjoys.”
Adolescent-Disliked (*n* = 20)			Parent-Disliked (*n* = 20)		
11 (55.0)	Not eating carbs	“How hard it is to not eat foods that I’m not allowed to eat.”	8 (40.0)	Limiting carbs/finding low carb options	“Hard to find low carb options.”
8 (40.0)	Hard to follow	“How hard it can be sometimes.”	7 (35.0)	*Nothing*	
7 (35.0)	Lack of food variety	“There is not a lot of big variety in foods you can eat.”	5 (25.0)	Lack of food variety	“Constantly looking for more (food) items so she is not eating the same thing all the time.”
6 (30.0)	*Nothing*		5 (25.0)	Too restrictive	“My daughter found it to be to restrictive and decided to give it up.”
5 (25.0)	Time	“Challenging with a busy schedule.”	5 (25.0)	Hard to follow	“Hard to stick to when we go through busy times.”
3 (15.0)	Eating with peers	“I do not enjoy watching my peers eating carb-filled lunch and snacks.”	5 (25.0)	Meal planning/preparation	“I need to learn how to make or get low carb meals to make ahead of time.”
Adolescent-Specifically made rPSMF difficult (*n* = 19)			Parent-Specifically made rPSMF difficult (*n* = 19)		
9 (47.4)	Not eating carbs	“Not being able to eat certain foods while others in my family are.”	9 (47.4)	Nothing	
7 (36.8)	Eating with peers	“Friends eating whatever they want.”	8 (42.1)	Food choices/variety	“The meal options are not customized to our exact preferences.”
6 (31.6)	*Nothing*		8 (42.1)	Time	“Time restraints.”
5 (26.3)	Food availability/variety	“Finding snack foods with low carbs.”	5 (26.3)	Cost	“Money for groceries.”
4 (21.1)	Time	“A busy scheduled makes the diet difficult to follow.”	4 (21.1)	Meal planning/preparation	“The biggest obstacle has been having time to meal prep.”
3 (15.8)	Special Events/Holidays/School	“I have a lot of special events.”	4 (21.1)	Limiting carbs	“It’s difficult because she misses bread, potatoes, and pasta.”
			3 (15.8)	Peer pressure	“It’s hard when she goes out with friends or when there are parties.”
